# Ethane-1,2-diaminium (*R*)-2-[4-(1-carboxyl­atoeth­oxy)phen­oxy]acetate

**DOI:** 10.1107/S1600536811054535

**Published:** 2011-12-23

**Authors:** Chang-Yue Ren, Guang-Feng Hou, Ying-Hui Yu, Jin-Sheng Gao

**Affiliations:** a Engineering Research Center of Pesticides of Heilongjiang University, Heilongjiang University, Harbin 150050, People’s Republic of China, and College of Chemistry and Materials Science, Heilongjiang University, Harbin 150080, People’s Republic of China

## Abstract

In the title compound, C_2_H_10_N_2_^2+^·C_11_H_10_O_6_^2−^, the two acetate groups of the cation form dihedral angles of 74.2 (4) and 63.9 (5)° with the central benzene ring. In the crystal, N—H⋯O hydrogen bonds link the cations and anions into layers parallel to the *ab* plane.

## Related literature

For the synthesis of the title chiral carb­oxy­lic acid, see: Bezwada *et al.* (2007 ▶). For the structure of a similar achiral carb­oxy­lic acid, see: Gong *et al.* (2010 ▶). 
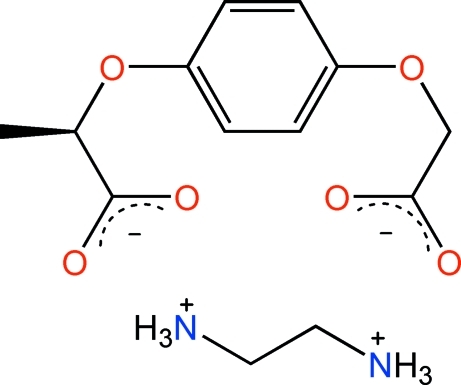


## Experimental

### 

#### Crystal data

C_2_H_10_N_2_^2+^·C_11_H_10_O_6_^2−^
*M* *_r_* = 300.31
Monoclinic, 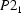

*a* = 10.066 (2) Å
*b* = 6.7887 (14) Å
*c* = 11.050 (2) Å
β = 99.30 (3)°
*V* = 745.2 (3) Å^3^
*Z* = 2
Mo *K*α radiation
μ = 0.11 mm^−1^
*T* = 293 K
0.62 × 0.10 × 0.06 mm

#### Data collection

Rigaku R-AXIS RAPID diffractometer
Absorption correction: multi-scan (*ABSCOR*; Higashi, 1995 ▶) *T*_min_ = 0.937, *T*_max_ = 0.994
7250 measured reflections
3288 independent reflections
1855 reflections with *I* > 2σ(*I*)
*R*_int_ = 0.066

#### Refinement

*R*[*F* ^2^ > 2σ(*F* ^2^)] = 0.066
*wR*(*F* ^2^) = 0.176
*S* = 1.01
3288 reflections
209 parameters
7 restraints
H atoms treated by a mixture of independent and constrained refinement
Δρ_max_ = 0.40 e Å^−3^
Δρ_min_ = −0.21 e Å^−3^

### 

Data collection: *RAPID-AUTO* (Rigaku, 1998 ▶); cell refinement: *RAPID-AUTO*; data reduction: *CrystalClear* (Rigaku/MSC, 2002 ▶); program(s) used to solve structure: *SHELXS97* (Sheldrick, 2008 ▶); program(s) used to refine structure: *SHELXL97* (Sheldrick, 2008 ▶); molecular graphics: *SHELXTL* (Sheldrick, 2008 ▶); software used to prepare material for publication: *SHELXL97*.

## Supplementary Material

Crystal structure: contains datablock(s) I, global. DOI: 10.1107/S1600536811054535/cv5213sup1.cif

Structure factors: contains datablock(s) I. DOI: 10.1107/S1600536811054535/cv5213Isup2.hkl

Supplementary material file. DOI: 10.1107/S1600536811054535/cv5213Isup3.cml

Additional supplementary materials:  crystallographic information; 3D view; checkCIF report

## Figures and Tables

**Table 1:** Hydrogen-bond geometry (Å, °)

*D*—H⋯*A*	*D*—H	H⋯*A*	*D*⋯*A*	*D*—H⋯*A*
N1—H33⋯O2^i^	0.90 (1)	1.86 (2)	2.736 (5)	164 (4)
N1—H32⋯O3	0.91 (1)	1.88 (1)	2.784 (5)	174 (5)
N1—H31⋯O3^ii^	0.90 (1)	1.94 (3)	2.764 (5)	150 (5)
N2—H34⋯O5	0.90 (1)	1.86 (2)	2.736 (5)	166 (5)
N2—H35⋯O5^iii^	0.90 (1)	1.94 (3)	2.748 (5)	148 (5)
N2—H36⋯O6^iv^	0.90 (1)	1.84 (1)	2.736 (5)	172 (5)

Symmetry codes: (i) 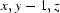; (ii) 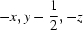; (iii) 
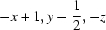
; (iv) 
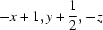
.

**Fig. 1.**
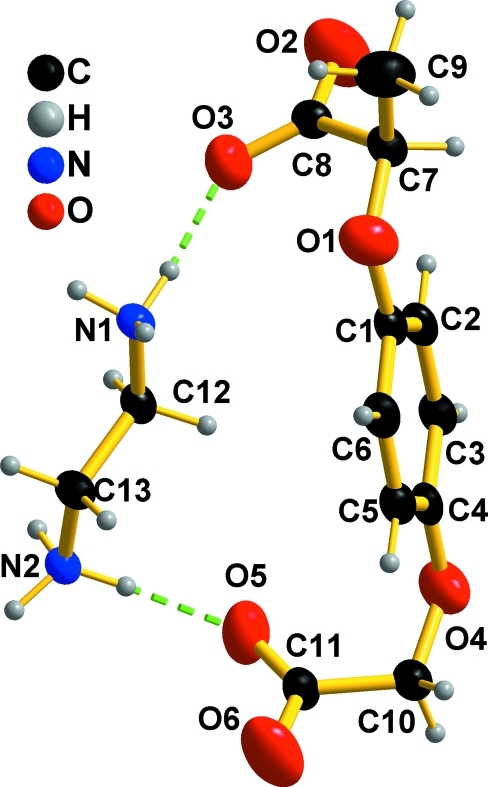
The molecular structure of the title compound, showing displacement ellipsoids at the 50% probability level for non-H atoms.

**Fig. 2.**
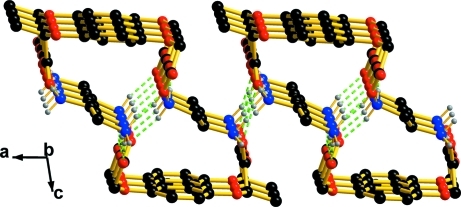
A partial packing view showing the intermolecular hydrogen bonds as dashed lines.

## supplementary crystallographic information

### Comment

The chiral ligands became one of the focus in supramolecular research due to their wide applications in catalytic and pharmaceutical industry. There are many reports about aromatic carboxylic acid, such as 4-carboxyphenoxyacetic acid (Gong *et al.*, 2010). However, structural reports about chiral carboxylic acids are rare. Herein, we report the synthesis and structure of a new chiral aromatic carboxylic acid derivative.

The asymmertric unit of title compound contains one (*R*)-2-(4-(1-carboxyethoxy)phenoxy)acetate anion and one ethane-1,2-diaminium cation (Fig. 1). Two acetate groups of the anion twist towards the same side of the benzenyl plane with the torsion angles of 105.8 (4) and 116.1 (5) °, respectively. A double layers structure parallel to the *ab* plane is built up by N—H···O hydrogen bonds linking the anions and cations (Fig. 2, Table 1).

### Experimental

(*R*)-2-(4-(carboxymethoxy)phenoxy)propanoic acid was prepared by the reaction of R-(+)-2-(4-hydroxy-phenoxy)propionic acid and methyl chloroacetate under alkaline condition (Bezwada *et al.*, 2007). (*R*)-2-(4-(carboxymethoxy)phenoxy)propanoic acid (0.048 g, 0.2 mmol) and ethylenediamine (1 mL, 0.2 mol / L) were dissolved in ethanol (15 mL). After stirring for 1 hour, the solution was filtered, and the filtrate was allowed to stand in a desiccator at room temperature for a few days. Colourless block crystals of title compound were obtained.

### Refinement

H atoms bound to C atoms were placed in calculated positions and treated as riding on their parent atoms, with C—H = 0.93 – 0.98 Å, and with *U*_iso_(H) = 1.2*U*_eq_(C). N-bound H atoms were located in a differece Fourier map and were refined with restraint N—H = 0.90 (1) Å, and *U*_iso_(H) = 1.5*U*_eq_(N). In the absence of any significant anomalous scatterers in the molecule, 1444 sets of Friedel pairs were merged before the final refinement and the absolute configuration was assigned to correspond with that of the known chiral centres in a precursor molecule, which remained unchanged during the synthesis of the title compound.

### Crystal data

**Table:**

C_2_H_10_N_2_^2+^·C_11_H_10_O_6_^2^^−^	*F*(000) = 320
*M**_r_* = 300.31	*D*_x_ = 1.338 Mg m^−^^3^
Monoclinic, *P*2_1_	Mo *K*α radiation, λ = 0.71073 Å
Hall symbol: P 2yb	Cell parameters from 4706 reflections
*a* = 10.066 (2) Å	θ = 3.0–27.6°
*b* = 6.7887 (14) Å	µ = 0.11 mm^−^^1^
*c* = 11.050 (2) Å	*T* = 293 K
β = 99.30 (3)°	Needle, colorless
*V* = 745.2 (3) Å^3^	0.62 × 0.10 × 0.06 mm
*Z* = 2	

### Data collection

**Table:**

Rigaku R-AXIS RAPID diffractometer	3288 independent reflections
Radiation source: fine-focus sealed tube	1855 reflections with *I* > 2σ(*I*)
graphite	*R*_int_ = 0.066
ω scan	θ_max_ = 27.5°, θ_min_ = 3.0°
Absorption correction: multi-scan (*ABSCOR*; Higashi, 1995)	*h* = −13→12
*T*_min_ = 0.937, *T*_max_ = 0.994	*k* = −8→8
7250 measured reflections	*l* = −14→14

### Refinement

**Table:**

Refinement on *F*^2^	Primary atom site location: structure-invariant direct methods
Least-squares matrix: full	Secondary atom site location: difference Fourier map
*R*[*F*^2^ > 2σ(*F*^2^)] = 0.066	Hydrogen site location: inferred from neighbouring sites
*wR*(*F*^2^) = 0.176	H atoms treated by a mixture of independent and constrained refinement
*S* = 1.01	*w* = 1/[σ^2^(*F*_o_^2^) + (0.081*P*)^2^] where *P* = (*F*_o_^2^ + 2*F*_c_^2^)/3
3288 reflections	(Δ/σ)_max_ < 0.001
209 parameters	Δρ_max_ = 0.40 e Å^−^^3^
7 restraints	Δρ_min_ = −0.21 e Å^−^^3^

### Special details

**Table:**

Geometry. All esds (except the esd in the dihedral angle between two l.s. planes) are estimated using the full covariance matrix. The cell esds are taken into account individually in the estimation of esds in distances, angles and torsion angles; correlations between esds in cell parameters are only used when they are defined by crystal symmetry. An approximate (isotropic) treatment of cell esds is used for estimating esds involving l.s. planes.
Refinement. Refinement of F^2^ against ALL reflections. The weighted R-factor wR and goodness of fit S are based on F^2^, conventional R-factors R are based on F, with F set to zero for negative F^2^. The threshold expression of F^2^ > 2sigma(F^2^) is used only for calculating R-factors(gt) etc. and is not relevant to the choice of reflections for refinement. R-factors based on F^2^ are statistically about twice as large as those based on F, and R- factors based on ALL data will be even larger.

### Fractional atomic coordinates and isotropic or equivalent isotropic displacement parameters (Å^2^)

**Table:**

	*x*	*y*	*z*	*U*_iso_*/*U*_eq_	
C1	0.2605 (4)	0.7401 (6)	0.3560 (4)	0.0435 (9)	
C2	0.3465 (5)	0.8903 (5)	0.3366 (4)	0.0501 (11)	
H2	0.3145	1.0187	0.3261	0.060*	
C3	0.4808 (4)	0.8504 (6)	0.3325 (4)	0.0505 (11)	
H3	0.5387	0.9525	0.3199	0.061*	
C4	0.5296 (4)	0.6592 (6)	0.3471 (4)	0.0481 (10)	
C5	0.4409 (4)	0.5082 (6)	0.3668 (4)	0.0508 (11)	
H5	0.4718	0.3790	0.3761	0.061*	
C6	0.3102 (5)	0.5494 (6)	0.3723 (4)	0.0506 (10)	
H6	0.2528	0.4481	0.3872	0.061*	
C7	0.0731 (4)	0.9589 (6)	0.3553 (4)	0.0491 (10)	
H7	0.1321	1.0400	0.4148	0.059*	
C8	0.0612 (4)	1.0535 (6)	0.2292 (4)	0.0462 (10)	
C9	−0.0588 (6)	0.9415 (10)	0.3946 (6)	0.0858 (17)	
H9A	−0.0492	0.8721	0.4712	0.129*	
H9B	−0.1191	0.8704	0.3337	0.129*	
H9C	−0.0945	1.0705	0.4045	0.129*	
C10	0.7100 (5)	0.4428 (7)	0.3237 (4)	0.0550 (11)	
H10A	0.6920	0.3635	0.3922	0.066*	
H10B	0.8068	0.4469	0.3267	0.066*	
C11	0.6471 (4)	0.3445 (6)	0.2061 (4)	0.0477 (10)	
C12	0.2339 (4)	0.5461 (6)	0.0488 (4)	0.0479 (10)	
H12A	0.3012	0.5738	0.1200	0.057*	
H12B	0.2407	0.6461	−0.0126	0.057*	
C13	0.2592 (4)	0.3446 (6)	−0.0019 (4)	0.0519 (11)	
H13A	0.2658	0.2478	0.0634	0.062*	
H13B	0.1838	0.3085	−0.0642	0.062*	
N1	0.0972 (3)	0.5515 (5)	0.0840 (3)	0.0455 (8)	
H31	0.025 (3)	0.541 (8)	0.024 (3)	0.068*	
H32	0.086 (5)	0.678 (3)	0.106 (4)	0.068*	
H33	0.066 (4)	0.455 (5)	0.128 (4)	0.068*	
N2	0.3835 (4)	0.3422 (5)	−0.0555 (3)	0.0475 (8)	
H34	0.446 (4)	0.367 (8)	0.010 (3)	0.071*	
H35	0.392 (5)	0.237 (5)	−0.103 (4)	0.071*	
H36	0.369 (5)	0.441 (5)	−0.111 (4)	0.071*	
O1	0.1245 (3)	0.7630 (4)	0.3592 (3)	0.0525 (8)	
O2	0.0518 (4)	1.2367 (4)	0.2264 (3)	0.0759 (11)	
O3	0.0581 (3)	0.9464 (4)	0.1361 (3)	0.0542 (7)	
O4	0.6629 (3)	0.6369 (4)	0.3379 (3)	0.0578 (8)	
O5	0.5970 (3)	0.4524 (4)	0.1170 (3)	0.0576 (8)	
O6	0.6516 (4)	0.1649 (5)	0.2056 (3)	0.0761 (11)	

### Atomic displacement parameters (Å^2^)

**Table:**

	*U*^11^	*U*^22^	*U*^33^	*U*^12^	*U*^13^	*U*^23^
C1	0.057 (3)	0.043 (2)	0.032 (2)	−0.006 (2)	0.0121 (18)	−0.0019 (17)
C2	0.075 (3)	0.0274 (19)	0.047 (3)	−0.0075 (18)	0.009 (2)	−0.0032 (16)
C3	0.058 (3)	0.044 (2)	0.051 (3)	−0.010 (2)	0.013 (2)	−0.008 (2)
C4	0.062 (3)	0.042 (2)	0.040 (2)	−0.007 (2)	0.005 (2)	−0.0107 (19)
C5	0.065 (3)	0.050 (3)	0.038 (2)	−0.001 (2)	0.011 (2)	0.0002 (18)
C6	0.068 (3)	0.040 (2)	0.045 (3)	−0.012 (2)	0.011 (2)	0.001 (2)
C7	0.057 (2)	0.047 (2)	0.048 (3)	−0.005 (2)	0.020 (2)	−0.002 (2)
C8	0.051 (2)	0.038 (2)	0.052 (3)	−0.004 (2)	0.014 (2)	0.0020 (19)
C9	0.085 (4)	0.080 (4)	0.100 (5)	0.011 (3)	0.038 (3)	0.022 (3)
C10	0.061 (3)	0.051 (2)	0.053 (3)	0.001 (2)	0.009 (2)	−0.005 (2)
C11	0.063 (3)	0.042 (2)	0.040 (3)	−0.006 (2)	0.013 (2)	0.001 (2)
C12	0.058 (3)	0.045 (2)	0.041 (2)	−0.003 (2)	0.0112 (19)	−0.004 (2)
C13	0.053 (3)	0.046 (2)	0.059 (3)	−0.0021 (19)	0.014 (2)	−0.009 (2)
N1	0.052 (2)	0.0373 (17)	0.048 (2)	0.0015 (17)	0.0123 (16)	−0.0024 (17)
N2	0.057 (2)	0.0417 (18)	0.043 (2)	0.0041 (18)	0.0071 (17)	−0.0041 (16)
O1	0.0629 (18)	0.0382 (14)	0.060 (2)	0.0010 (14)	0.0213 (15)	0.0086 (13)
O2	0.118 (3)	0.0369 (17)	0.079 (3)	−0.0101 (18)	0.033 (2)	0.0051 (15)
O3	0.0700 (19)	0.0511 (16)	0.0407 (18)	0.0024 (15)	0.0066 (14)	−0.0018 (14)
O4	0.0515 (17)	0.0547 (18)	0.067 (2)	−0.0070 (15)	0.0098 (15)	−0.0218 (15)
O5	0.076 (2)	0.0496 (16)	0.0442 (18)	−0.0125 (16)	0.0013 (15)	0.0015 (15)
O6	0.125 (3)	0.0359 (17)	0.072 (2)	−0.0122 (19)	0.027 (2)	−0.0036 (15)

### Geometric parameters (Å, °)

**Table:**

C1—C2	1.377 (6)	C9—H9C	0.9600
C1—O1	1.384 (5)	C10—O4	1.418 (5)
C1—C6	1.389 (6)	C10—C11	1.507 (6)
C2—C3	1.387 (6)	C10—H10A	0.9700
C2—H2	0.9300	C10—H10B	0.9700
C3—C4	1.387 (6)	C11—O6	1.221 (5)
C3—H3	0.9300	C11—O5	1.264 (5)
C4—O4	1.371 (5)	C12—N1	1.489 (5)
C4—C5	1.399 (6)	C12—C13	1.516 (5)
C5—C6	1.356 (6)	C12—H12A	0.9700
C5—H5	0.9300	C12—H12B	0.9700
C6—H6	0.9300	C13—N2	1.468 (6)
C7—O1	1.426 (5)	C13—H13A	0.9700
C7—C9	1.467 (6)	C13—H13B	0.9700
C7—C8	1.522 (6)	N1—H31	0.904 (10)
C7—H7	0.9800	N1—H32	0.907 (10)
C8—O2	1.247 (5)	N1—H33	0.900 (10)
C8—O3	1.256 (5)	N2—H34	0.898 (10)
C9—H9A	0.9600	N2—H35	0.900 (10)
C9—H9B	0.9600	N2—H36	0.900 (10)
			
C2—C1—O1	124.8 (4)	O4—C10—H10A	108.8
C2—C1—C6	119.2 (4)	C11—C10—H10A	108.8
O1—C1—C6	116.0 (4)	O4—C10—H10B	108.8
C1—C2—C3	120.1 (4)	C11—C10—H10B	108.8
C1—C2—H2	120.0	H10A—C10—H10B	107.7
C3—C2—H2	120.0	O6—C11—O5	125.9 (4)
C2—C3—C4	120.5 (4)	O6—C11—C10	115.8 (4)
C2—C3—H3	119.7	O5—C11—C10	118.3 (4)
C4—C3—H3	119.7	N1—C12—C13	109.6 (3)
O4—C4—C3	115.3 (4)	N1—C12—H12A	109.7
O4—C4—C5	126.0 (4)	C13—C12—H12A	109.7
C3—C4—C5	118.8 (4)	N1—C12—H12B	109.7
C6—C5—C4	120.2 (4)	C13—C12—H12B	109.7
C6—C5—H5	119.9	H12A—C12—H12B	108.2
C4—C5—H5	119.9	N2—C13—C12	111.3 (3)
C5—C6—C1	121.2 (4)	N2—C13—H13A	109.4
C5—C6—H6	119.4	C12—C13—H13A	109.4
C1—C6—H6	119.4	N2—C13—H13B	109.4
O1—C7—C9	104.9 (4)	C12—C13—H13B	109.4
O1—C7—C8	113.3 (4)	H13A—C13—H13B	108.0
C9—C7—C8	111.3 (4)	C12—N1—H31	119 (3)
O1—C7—H7	109.0	C12—N1—H32	105 (3)
C9—C7—H7	109.0	H31—N1—H32	99 (4)
C8—C7—H7	109.0	C12—N1—H33	122 (3)
O2—C8—O3	124.5 (4)	H31—N1—H33	91 (4)
O2—C8—C7	116.0 (4)	H32—N1—H33	118 (5)
O3—C8—C7	119.6 (4)	C13—N2—H34	102 (3)
C7—C9—H9A	109.5	C13—N2—H35	114 (3)
C7—C9—H9B	109.5	H34—N2—H35	120 (5)
H9A—C9—H9B	109.5	C13—N2—H36	102 (3)
C7—C9—H9C	109.5	H34—N2—H36	115 (5)
H9A—C9—H9C	109.5	H35—N2—H36	102 (4)
H9B—C9—H9C	109.5	C1—O1—C7	117.4 (3)
O4—C10—C11	113.9 (4)	C4—O4—C10	117.4 (3)

### Hydrogen-bond geometry (Å, °)

**Table:**

*D*—H···*A*	*D*—H	H···*A*	*D*···*A*	*D*—H···*A*
N1—H33···O2^i^	0.90 (1)	1.86 (2)	2.736 (5)	164 (4)
N1—H32···O3	0.91 (1)	1.88 (1)	2.784 (5)	174 (5)
N1—H31···O3^ii^	0.90 (1)	1.94 (3)	2.764 (5)	150 (5)
N2—H34···O5	0.90 (1)	1.86 (2)	2.736 (5)	166 (5)
N2—H35···O5^iii^	0.90 (1)	1.94 (3)	2.748 (5)	148 (5)
N2—H36···O6^iv^	0.90 (1)	1.84 (1)	2.736 (5)	172 (5)

Symmetry codes: (i) *x*, *y*−1, *z*; (ii) −*x*, *y*−1/2, −*z*; (iii) −*x*+1, *y*−1/2, −*z*; (iv) −*x*+1, *y*+1/2, −*z*.
